# Durable Hydrophobic Iridescent Films with Tunable Colors from Self‐Assembled Cellulose Nanocrystals

**DOI:** 10.1002/smll.202409701

**Published:** 2024-12-24

**Authors:** Zongzhe Li, Phoebe Wang, Yinghao Zhang, Carl A. Michal, Mark J. MacLachlan

**Affiliations:** ^1^ Department of Chemistry University of British Columbia 2036 Main Mall Vancouver BC V6T 1Z1 Canada; ^2^ Department of Physics and Astronomy The University of British Columbia 6224 Agricultural Rd. Vancouver BC V6T 1Z1 Canada; ^3^ Stewart Blusson Quantum Matter Institute University of British Columbia 2355 East Mall Vancouver BC V6T 1Z4 Canada; ^4^ WPI Nano Life Science Institute Kanazawa University Kanazawa 920–1192 Japan; ^5^ Bioproducts Institute University of British Columbia 2360 East Mall Vancouver BC V6T 1Z3 Canada

**Keywords:** cellulose nanocrystal, chiral nematic, hydrophobic, iridescent, photonic material, self‐assembly

## Abstract

Cellulose nanocrystals (CNCs) are known to self‐assemble into a left‐handed chiral nematic lyotropic liquid crystalline phase in water. When captured in the solid state, this structure can impart films with photonic properties that make them promising candidates in photonics, sensing, security, and other areas. Unfortunately, the intrinsic hydrophilicity of CNCs renders these iridescent films susceptible to moisture, thereby limiting their practicality. To address this issue, a novel strategy to prepare hydrophobic iridescent films from pre‐assembled CNC films is reported here. These films underwent a swelling process, followed by esterification using acid anhydrides to render them hydrophobic. By increasing the alkyl chain length of the anhydride reagent, the hydrophobicity of the resulting iridescent films can be enhanced. They showed water contact angles ranging from 34° to 115° and demonstrated tunable structural color spanning from blue to red. Moreover, they also exhibited good durability when exposed to water for 24 h. This innovative method for producing durable hydrophobic iridescent thin films is expected to facilitate their use in water‐proof photonic coatings, optical sensors, and other applications.

## Graphical abstract

Durable hydrophobic iridescent films are developed from self‐assembled chiral nematic cellulose nanocrystals. They present tunable structural colors and hydrophobicity by using acid anhydrides with various alkyl chain lengths for functionalization, both of which remain intact even after exposure to water. This novel strategy to fabricate hydrophobic iridescent films can expedite their implementation as waterproof photonic materials.

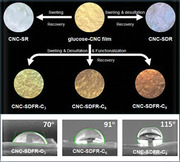

## 1 Introduction

Cellulose is the most abundant biopolymer on earth, widely found in plants and tunicates. Acid hydrolysis of biomass products, such as wood pulp,^[^ ^1^ ^]^ can yield cellulose nanocrystals (CNCs), a rigid, rod‐shaped nanomaterial with a high aspect ratio.^[^ ^2^ ^]^ When using sulfuric acid for hydrolysis, the resulting CNCs are functionalized with sulfate half‐ester groups that impart a surface charge on the particles and enable them to form stable colloidal suspensions in water.^[^ ^3^ ^]^ Due to their biocompatibility, biodegradability, and renewability, CNCs have been explored in food packaging,^[^ ^4^, ^5^ ^]^ drug delivery,^[^ ^6^, ^7^ ^]^ and biosensing.^[^ ^8^, ^9^ ^]^ Furthermore, in aqueous suspensions, CNCs spontaneously self‐assemble into a left‐handed chiral nematic (cholesteric) liquid crystalline phase.^[^ ^10^, ^11^, ^12^ ^]^ This chiral nematic structure can be retained during solvent evaporation, resulting in iridescent CNC films, where the highly ordered nanostructure of CNCs behaves as a photonic crystal.^[^ ^13^ ^]^ These nanomaterials with unique chiral nematic structures have been investigated as soft and hard templates for functional chiral photonic materials.^[^ ^14^, ^15^ ^]^ Given their fascinating chiral photonic properties, numerous research groups have also been working on developing advanced materials derived from iridescent CNC thin films.^[^ ^16^, ^17^, ^18^, ^19^ ^]^ Moreover, Vignolini et al. have pioneered the fabrication of large‐scale CNC photonic films for practical applications.^[^ ^20^ ^]^ However, the abundant hydroxyl and sulfate half‐ester groups on the CNC surfaces make these materials inherently hydrophilic. This poses a practical problem in that photonic materials are usually susceptible to swelling and damage from water and steam, which can disrupt their photonic properties, thus limiting their applicability under moist conditions.^[^ ^21^, ^22^ ^]^

Numerous methods have been reported to prepare hydrophobic CNC films, including plasma fluorination,^[^ ^23^ ^]^ acylation,^[^ ^24^, ^25^ ^]^ silanization,^[^ ^26^ ^]^ and esterification.^[^ ^27^ ^]^ However, most of these approaches directly modify individual, colloidal CNC particles, substituting their surface hydroxyl groups and inhibiting their self‐assembly into a chiral nematic structure afterward.^[^ ^28^ ^]^ Therefore, the resulting films do not display any structural color and are ineffective as photonic materials.^[^ ^29^, ^30^, ^31^, ^32^ ^]^ On the other hand, hydrophobic iridescent CNC films have rarely been reported before.^[^ ^22^ ^]^ Most of those few reports use direct surface coating of the CNC films to increase their surface hydrophobicity.^[^ ^33^, ^34^, ^35^ ^]^ Unfortunately, these materials remain vulnerable to abrasion. If the surface coating is scraped during use, the material will be susceptible to moisture. More importantly, the single thin layer of physical or chemical coating may not resist water for an extended period of time, limiting the material's durability and practicality. Therefore, it is essential to have the entire hydrophobic iridescent film solely composed of hydrophobically modified CNCs arranged with chiral nematic order. To the best of our knowledge, this has never been achieved before.

Here, to address this challenge, a new strategy has been developed for the in situ functionalization of the CNCs with chiral nematic order from swollen pre‐assembled CNC films. This approach allows us to circumvent the failure of CNCs to form chiral nematic phases after functionalization. Following solvent exchange and solvent evaporation, a series of hydrophobic iridescent CNC films were readily prepared. Acid anhydrides were chosen over other esterification agents for CNC modification due to their higher grafting efficiencies.^[^ ^36^ ^]^ As demonstrated by water contact angle analysis, the hydrophobicity of the iridescent films can be adjusted by varying the acid anhydride being used. Homogeneous functionalization of the films was verified using multiple analytical techniques, including scanning electron microscopy – energy dispersive X‐ray (SEM‐EDX) analysis, thermogravimetric analysis (TGA), Fourier transform infrared (FTIR) spectroscopy, and ^13^C solid‐state cross‐polarization magic angle spinning (CP‐MAS) nuclear magnetic resonance (NMR) spectroscopy. UV–vis spectroscopy showed that the structural color of these hydrophobic films can be systematically tuned from blue to red using acid anhydrides with longer alkyl chains. Circular dichroism (CD) spectroscopy and SEM structural analysis also revealed that the chiral nematic structure of CNCs is retained after the functionalization and is responsible for the iridescent structural color. Moreover, benefiting from their thoroughly hydrophobic characteristic, these materials also presented excellent durability in water to retain their hydrophobicity and color integrity. These results provide solid evidence for the effectiveness of this new method to prepare durable hydrophobic iridescent thin films with tunable colors as potential water‐proof photonic materials.

## 2 Results and Discussion

**Scheme 1:**
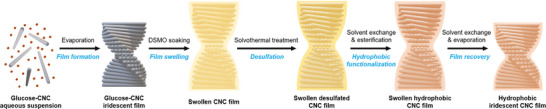
Illustration of the five‐step strategy to prepare hydrophobic iridescent CNC films.

The strategy we developed for the preparation of hydrophobic iridescent CNC films involves five steps: film formation, swelling, solvothermal desulfation, functionalization, and recovery (**Scheme** **1**). In the first step, iridescent glucose‐CNC (g‐CNC) films with chiral nematic structure are prepared by adding glucose into aqueous CNC suspensions, followed by evaporation of the solvent. In the following swelling step, glucose acts as a sacrificial agent to assist the infiltration of DMSO into the CNC matrix.^[^ ^37^ ^]^ Compared to CNC films, the swollen CNC chiral nematic matrix provides larger gaps in between individual CNCs, offering access for effective functionalization. Subsequently, a solvothermal treatment is employed to desulfate the CNCs.^[^ ^38^ ^]^ During this process, sulfate half‐ester groups on the CNC surfaces were substituted by hydroxyl groups. This reduces the density of charged groups on the surface and increases the hydrogen bonding interactions between adjacent CNCs, thus helping to enhance the stability of the materials. Finally, after the esterification process using acid anhydrides, the functionalized swollen CNC chiral nematic matrices are recovered as hydrophobic iridescent CNC films through solvent exchange and evaporation.

### 2.1 Effectiveness of Solvothermal Treatment

**Figure 1:**
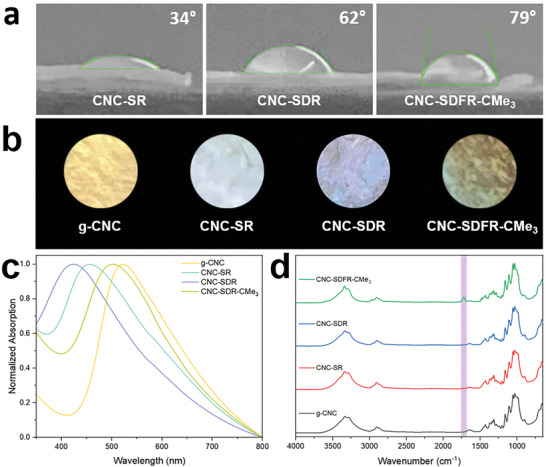
a) Photographs showing surface water contact angle measurements of CNC‐SR, CNC‐SDR, and CNC‐SDFR‐CMe_3_. b) Photographs, c) UV–vis spectra, and d) FTIR spectra of g‐CNC, CNC‐SR, CNC‐SDR and CNC‐SDFR‐CMe_3_, respectively. The purple band highlights the C═O stretching mode of the ester group after esterification. The samples shown in (b) are 10 mm in diameter. The photographs shown in (b) were obtained under ambient light without polarizers, then cropped into a circular shape and superposed on a black background. Samples were placed on black cloth for photography.

To demonstrate the effectiveness of solvothermal desulfation, CNC‐SR (recovered from swollen CNC film) and CNC‐SDR (recovered from desulfated CNC film) were prepared separately. As shown by the water contact angle analysis, the surface contact angle of CNC‐SR is 34°, while with an additional solvothermal treatment step after swelling, CNC‐SDR exhibits a significant increase in the surface contact angle to 62° (**Figure** **1**a). This suggests an improved hydrophobicity of CNC‐SDR in comparison to CNC‐SR, indicating the effectiveness of the solvothermal treatment. Moreover, the UV–vis spectra show a blue shift in structural color going from g‐CNC to CNC‐SR to CNC‐SDR (Figure 1c). The blue shift of ≈70 nm observed for CNC‐SR relative to g‐CNC can be attributed to the loss of glucose, resulting in a shorter distance between CNCs. Subsequent desulfation results in decreased electrostatic repulsion between the sulfate half‐ester groups, leading to further compression of the chiral nematic structure and causing an even more blue‐shifted structural color for CNC‐SDR (ca. 100 nm compared to g‐CNC).

### 2.2 Effectiveness of Esterification

After the swelling and solvothermal desulfation of g‐CNC film, the functionalization of the CNC film was performed through esterification using acid anhydrides. To demonstrate the effectiveness of our strategy, a bulky acid anhydride, trimethylacetic anhydride, was chosen for the first trial. FTIR analysis of the resulting CNC‐SDFR‐CMe_3_ film shows a new peak at ca. 1720 cm^−1^, corresponding to the C═O stretching from the ester, confirming the successful grafting of the trimethylacetate groups onto the CNCs (Figure 1d). The hydrophobicity of the resulting CNC‐SDRF‐CMe_3_ film was validated by surface contact angle measurements, demonstrating a notable increase (to 79°) compared to CNC‐SDR (Figure 1a). Moreover, a red‐shifted structural color was observed for CNC‐SDFR‐CMe_3_ compared to CNC‐SDR, which was also revealed by UV–vis spectroscopy (Figure 1b,c). We attribute this to the grafted alkyl chains on the CNC surfaces, which increase the distance between the CNCs, expanding the helical pitch of the chiral nematic structure and red‐shifting the structural color.

**Figure 2:**
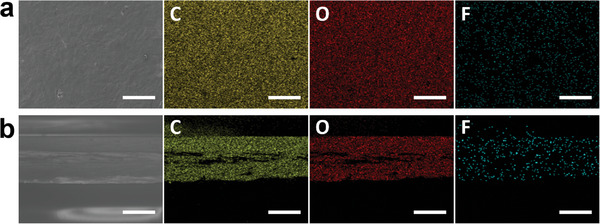
SEM images of a) surface and b) cross‐section of CNC‐SDFR‐CF_3_. To the right of each image is the SEM‐EDX mapping data showing the distribution of C, O, and F. (Scale bar: 250 nm).

Furthermore, to evaluate the effectiveness of the functionalization strategy throughout the material, trifluoroacetic anhydride was used to introduce CF_3_ groups into the CNC film (CNC‐SDFR‐CF_3_). As illustrated in **Figure** **2**, EDX mapping of the CNC‐SDFR‐CF_3_ film shows that carbon, oxygen, and fluorine are homogeneously distributed both on the surface and in the cross‐section. These results provide evidence that fluorine is uniformly distributed throughout the entire material, and the functionalization is not limited to the surface of the CNC film, but rather effective throughout the entire material.

### 2.3 Effects of the Alkyl Chain Length of Acid Anhydride

As the length of alkyl groups grafted to CNCs is known to affect the hydrophobicity of the resulting materials,^[^ ^39^ ^]^ we explored the effects of alkyl chain length of acid anhydrides used for CNC esterification. Hydrophobic CNC films were prepared with three different acid anhydrides (butyric, heptanoic, and decanoic anhydride), in which the number of carbon atoms in the alkyl chain increases in an arithmetic progression; the resulting films are named CNC‐SDFR‐C_3_, CNC‐SDFR‐C_6_, and CNC‐SDFR‐C_9_, respectively.

**Figure 3:**
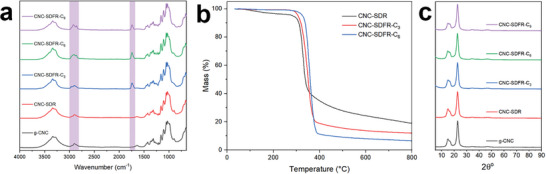
a) FTIR spectra (highlighted regions indicate where new peaks appear), b) TGA curves (10 °C min^−1^, under N_2_), and c) PXRD diffractograms of CNC films before and after esterification with acid anhydrides.

Characterization via FTIR spectroscopy confirmed the successful grafting of alkyl chains onto the functionalized CNC films, as evidenced by the appearance of a C═O stretching mode at ca. 1740 cm^−1^ from the ester group, and C─H stretching at 2925 and 2850 cm^−1^, from the methylene and methyl groups on the alkyl chains, respectively (**Figure** **3**a). Elemental analysis results also show higher carbon and hydrogen content in the functionalized films with longer alkyl chains (Table S1, Supporting Information). However, the degree of substitution (DS) decreases from 0.30 to 0.19 with the increase of the alkyl chain length from CNC‐SDFR‐C_3_ to CNC‐SDFR‐C_9_. TGA was conducted to examine the thermal stability of the CNC films (Figure 3b). The loss of weight below ≈300 °C can be attributed to the loss of adsorbed water and is only observed in the unfunctionalized CNC film. In contrast, the functionalized CNC films show no weight loss within this range, indicating no adsorbed water and illustrating their hydrophobic characteristic. Furthermore, the results demonstrate that the thermal stability of the material substantially increases with the introduction of longer alkyl chains, possibly attributed to the formation of large hydrophobic shields that can protect the CNCs from species that catalyze their thermal degradation.^[^ ^40^ ^]^

**Figure 4:**
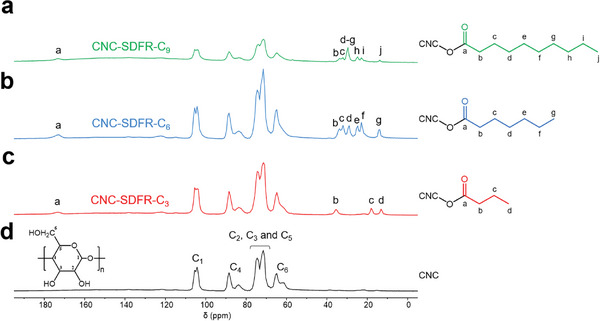
^13^C Solid‐state ^1^H‐decoupled CP‐MAS NMR spectra of the a–c) functionalized and d) desulfated CNC films, with the assignment of the characteristic signals.

Solid‐state ^13^C NMR spectroscopy was also performed to prove the successful esterification. As depicted in **Figure** **4**, the ^13^C CP‐MAS NMR spectra show all of the resonances expected for the carbons of cellulose at 105 ppm (C_1_), 89 ppm (C_4_ crystalline), 84 ppm (C_4_ amorphous), 70–75 ppm (C_2_, C_3,_ and C_5_), 65 ppm (C_6_ crystalline), and 62 ppm (C_6_ amorphous) (Figure 4d),^[^ ^41^ ^]^ while the peaks for grafted alkyl chains in the functionalized films appear at 175 ppm (carbonyl) and at 10–40 ppm (methylene and methyl groups) (Figure 4a–c). The crystalline nature of the functionalized CNCs was also validated through powder X‐ray diffraction (PXRD) analysis, as consistent patterns with characteristic CNC peaks at 16.5° and 22.5° were observed in all CNC films pre‐ and post‐functionalization (Figure 3c).

**Figure 5:**
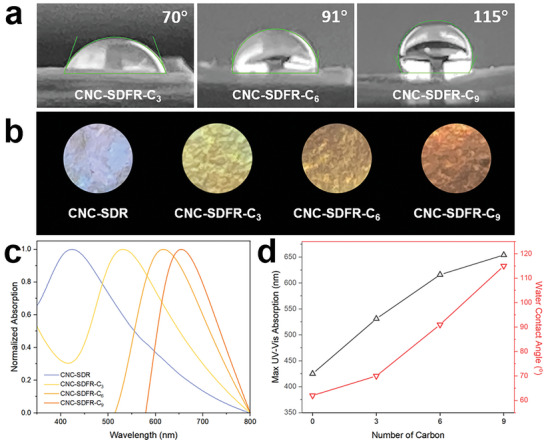
a) Photographs showing surface water contact angle measurements of CNC‐SDFR‐C_3_, CNC‐SDFR‐C_6_, and CNC‐SDFR‐C_9_. b) Photographs and c) UV–vis spectra of CNC‐SDR, CNC‐SDFR‐C_3_, CNC‐SDFR‐C_6,_ and CNC‐SDFR‐C_9_, respectively. d) Changing trend of maximum UV–vis absorption and water contact angle versus the alkyl chain length. The samples shown in (b) are 10 mm in diameter. The photographs shown in (b) were obtained under ambient light without polarizers, then cropped into a circular shape and superposed on a black background. Samples were placed on black cloth for photography.

The functionalized CNC films (CNC‐SDFR‐C_3_, CNC‐SDFR‐C_6_, and CNC‐SDFR‐C_9_) showed progressively higher contact angles (and therefore increasing hydrophobicity) with longer alkyl chains grafted onto the CNCs. For example, CNC‐SDFR‐C_3_ had a surface contact angle of 70°, whereas CNC‐SDFR‐C_9_ had a surface contact angle of 115° (**Figure** **5**a). This value is not only superior to the previously reported hydrophobic iridescent CNC films,^[^ ^22^ ^]^ but is also comparable to hydrophobic CNC films with no iridescence prepared using various methods.^[^ ^24^, ^25^, ^26^, ^27^ ^]^

Furthermore, as with CNC‐SDFR‐CMe_3_, all three functionalized CNC films displayed a red‐shifted structural color compared to CNC‐SDR, which was also characterized by UV–vis spectra (Figure 5b,c). It is noteworthy that the extent of red‐shifting in the iridescence of the functionalized CNC films correlates with an increase in the alkyl chain length of the acid anhydride used. This can be rationalized by the increasing distance between adjacent CNCs grafted with longer alkyl chains, which results in a longer helical pitch of the CNC chiral nematic structure after film recovery. In conclusion, both the maximum UV–vis absorption and water contact angle increase along with the increase of the alkyl chain length (Figure 5d).

### 2.4 Durability and Solvent Absorption Analysis

**Figure 6:**
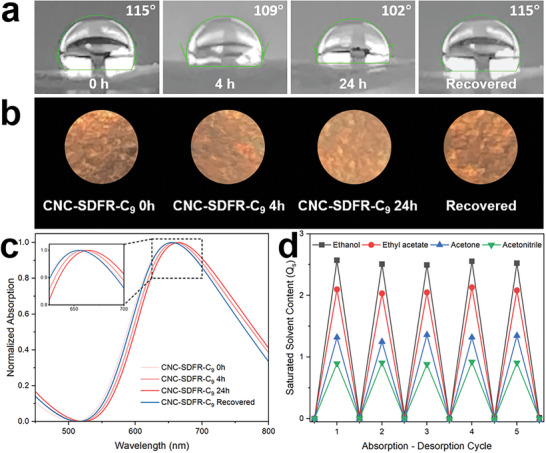
a) Photographs showing surface water contact angle, b) photographs, and c) UV–vis spectra of CNC‐SDFR‐C_9_ at different stages. d) Solvent absorption–desorption test results of CNC‐SDFR‐C_9_ with ethanol, ethyl acetate, acetone, and acetonitrile. The samples shown in (b) are 10 mm in diameter. The photographs shown in (b) were obtained under ambient light without polarizers, then cropped into a circular shape and superposed on a black background. Samples were placed on black cloth for photography.

As CNC‐SDFR‐C_9_ showed the greatest hydrophobicity (i.e., highest water surface contact angle), a time‐lapse study was conducted to evaluate its resistance to water. The film was immersed in water for 4 and 24 h to simulate short‐term and long‐term exposure, respectively. As depicted in **Figure** **6**a, over a period of 24 h, CNC‐SDFR‐C_9_ retained its hydrophobicity, with only a small decrease in the surface contact angle from 115° to 102°, and no distinguishable change in its structural color (Figure 6b). In contrast, most of the previously reported hydrophobic CNC films showed a significant decrease (∼20%) in water contact angle within only 2–3 min.^[^ ^22^, ^42^, ^43^ ^]^ This durability of the hydrophobic iridescent films is attributed to their thoroughly hydrophobic nature, which results from the elaborate design of our functionalization strategy. Moreover, the UV–vis spectra at all four stages (0, 4, 24 h, and recovered) are almost identical, with only slight redshifts (ca. 7 and 11 nm) at the stages after soaking in water for 4 and 24 h, and recovered to its original state after drying (Figure 6c). FTIR spectra of the sample before and after soaking in water for 24 h showed no significant differences, suggesting no water absorption into the film after long‐term exposure to water (Figure S3, Supporting Information). These results demonstrate the high color stability of the hydrophobic iridescent films in water over extended periods and affirm the effectiveness of our functionalization strategy in preserving the hydrophobic properties of the resulting photonic films.

Grafting of long alkyl chains onto CNCs is a well‐established method for enhancing their solvent uptake capacity.^[^ ^44^ ^]^ To ascertain the potential applications of these materials for solvent absorption, we conducted solvent absorption‐desorption tests on CNC‐SDFR‐C_9_ over a total of five cycles. In contrast to unfunctionalized CNC materials,^[^ ^45^ ^]^ the hydrophobic CNC films showed consistent solvent absorption performance throughout all five cycles (Figure 6d), indicating their potential as reusable solvent absorbers. Moreover, it is noteworthy that, unlike common aerogel‐based solvent absorbers, CNC‐SDFR‐C_9_ became colorless after saturation with solvents (Figure S4, Supporting Information), suggesting its potential as a solvent sensor or saturation indicator.

### 2.5 Structural Analysis of the Hydrophobic Iridescent CNC Films

**Figure 7:**
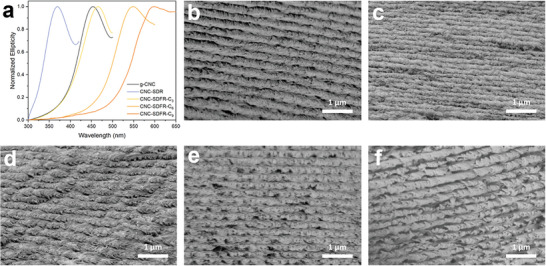
a) CD spectra and b–f) cross‐sectional SEM images of g‐CNC, CNC‐SDR, CNC‐SDFR‐C_3_, CNC‐SDFR‐C_6_, and CNC‐SDFR‐C_9_.

The presence of chiral nematic microstructure in the desulfated and functionalized CNC films was determined by both CD spectroscopy and SEM analysis. Peaks with positive ellipticity observed in the CD spectra correspond to the reflection of left‐handed circularly polarized light, thereby confirming the preservation of the chiral nematic structure after desulfation and esterification (Figure 7a). Moreover, the observed trend in the CD spectra agrees well with that observed in the UV–vis spectra, where the structural color of the CNC films exhibits a blue shift after desulfation and a red shift after functionalization with various acid anhydrides. The twisted layered structure, which is characteristic of the left‐handed chiral nematic ordering of CNCs, could be observed in the cross‐sectional SEM image of g‐CNC (Figure 7b). Notably, this unique chiral nematic structure is retained following desulfation and functionalization processes (Figure 7c–f), providing direct evidence that the structural color of the film originates from its chiral nematic microstructure. The half pitches of these films were also measured (*n* > 12) to be 150 ± 12, 237 ± 26, 259 ± 15, and 292 ± 16 nm, from CNC‐SDR to CNC‐SDFR‐C_9_, respectively. This increasing trend in helical pitch with the increase of the alkyl chain length is in agreement with our observations of the red‐shifting effect and supports the expansion of the helical pitch by using acid anhydride with longer alkyl chain length being responsible for their different structural colors.

## 3 Conclusion

Highly durable hydrophobic iridescent films with tunable colors were prepared based on pre‐assembled chiral nematic glucose‐cellulose nanocrystal (g‐CNC) films through esterification using acid anhydride reagents. The resulting films show tunable hydrophobicity with water contact angles ranging from 34° to 115°, and structural colors across the visible spectrum. These properties can be modulated by varying the alkyl chain length of the employed acid anhydrides. These materials also show excellent durability as their hydrophobicity remained even after soaking in water for 24 h, with no change in their photonic properties. SEM‐EDX, FTIR, TGA, and ^13^C CP‐MAS NMR analyses demonstrated that the functionalization extends throughout the entire CNC film rather than being confined to the surface. The chiral nematic structure of CNCs also remains intact following solvothermal treatment and functionalization steps and is responsible for the iridescent structural colors. It is anticipated that this novel strategy to prepare durable hydrophobic iridescent films with tunable colors will accelerate their application as water‐insensitive photonic materials.

## 4 Experimental Section

### Material

All chemicals were purchased from standard suppliers and used without further purification. The CNC aqueous suspensions were provided by FPInnovations (CNC‐H^+^, 4.0 wt %, pH = 2.1, conductivity = 1759.4 µS cm^−1^, zeta potential = – 52.9 mV, average effective hydrodynamic radius (DLS) = 96.2 ± 0.7 nm), which were prepared from kraft pulp using a previously reported procedure.^[^ ^46^ ^]^

### Preparation of Chiral Nematic Glucose‐CNC (g‐CNC) Films

D‐Glucose (100 mg) was added to CNC aqueous suspension (4.0 wt %, pH = 2.4, 20 g) and the mixture was sonicated for 10 min in a sonicator bath to fully dissolve the glucose. The obtained suspension was cast in a 55 mm diameter polystyrene Petri dish and then allowed to evaporate under ambient conditions (room temperature and ca. 50% RH). Once the film had dried, as indicated by the uniformity of its structural coloration, it was stored inside a desiccator at room temperature with ca. 75% RH for further experiments. UV–vis spectroscopy showed the resulting g‐CNC film has a maximum reflection at 523 nm (Figure S1, Supporting Information).

### Glucose‐CNC (g‐CNC) Film Swelling & Desulfation

A square piece of g‐CNC film (1 × 1 cm^2^) in a cut vial (20 mm diameter, 26 mm height) was bathed in 1 mL of DMSO to initiate the swelling process (Figure S2, Supporting Information). The vial was swirled for 2 min to suspend the film in DMSO and prevent the adhesion of the film to the bottom of the vial. After 40 min of swelling, solvothermal treatment was conducted based on a reported procedure.^[^ ^38^ ^]^ The vial containing the swollen CNC film was transferred into a Teflon‐lined stainless‐steel autoclave, which was then sealed and placed into a programmable oven (Thermolyne, Type 47900). The sample was heated to 120 °C over 1 h and was maintained at this temperature for another 20 h. The system was programmed to gradually cool down to room temperature over 20 min to complete the solvothermal treatment. Subsequently, the desulfated CNC film was transferred into a 20 mL scintillation vial, and solvent exchanged successively with DMSO (once), H_2_O (3 times), and EtOH (3 times) at 30 min intervals. Afterward, the swollen film was stored in EtOH for further experiments.

### Hydrophobic Functionalization

The swollen desulfated CNC film was functionalized using various acid anhydrides, including trimethylacetic anhydride, trifluoroacetic anhydride, butyric anhydride, heptanoic anhydride and decanoic anhydride. The resulting films were named as CNC‐SDFR‐CMe_3_, CNC‐SDFR‐CF_3_, CNC‐SDFR‐C_3_, CNC‐SDFR‐C_6_ and CNC‐SDFR‐C_9_, respectively. Prior to functionalization, the swollen CNC film stored in EtOH was solvent exchanged 3 times with pyridine at 30 min intervals. Subsequently, the swollen film was transferred into a new 20 mL scintillation vial, followed by addition of 4 mL of the desired acid anhydride. The vial was then tightly sealed and heated to 80 °C for 24 h in an oil bath to functionalize the film. It should be noted that due to the lower boiling point of trifluoroacetic anhydride, its esterification process was conducted using a 0.1 m solution in acetonitrile, and heated to 50 °C for 24 h.

### CNC Film Recovery

Following the esterification, the swollen functionalized CNC film was subsequently solvent exchanged with EtOH for 3 times. After the final solvent exchange, excess EtOH was removed and the swollen film was air‐dried in a fume hood to recover the hydrophobic iridescent CNC film.

### Preparation of CNC‐SR

A square piece of g‐CNC film (1 × 1 cm^2^) was swollen following the same procedure mentioned previously, and then directly solvent exchanged with EtOH for 3 times and recovered as CNC‐SR.

### Preparation of CNC‐SDR

A square piece of g‐CNC film (1 × 1 cm^2^) was swollen and desulfated following the same procedure mentioned previously, and then directly solvent exchanged with EtOH for 3 times and recovered as CNC‐SDR.

### Characterization

SEM images were collected using a Zeiss Crossbeam XB350 CryoFIB‐SEM electron microscope, with 2 nm of Au sputter‐coated on all of the samples before imaging. SEM‐EDX was also performed using the same SEM microscope, with an Oxford XMAX 170 mm EDX detector equipped for the EDX mapping.

Dynamic light scattering (DLS) measurements were carried out in triplicate using a NanoBrook Omni particle size analyzer (Brookhaven).

UV–vis spectroscopy was conducted in transmission mode using a Cary 5000 UV–vis–NIR spectrometer (Agilent).

CD spectroscopy was conducted in reflection mode using a J‐815 CD Spectrometer (Jasco), with the sample directly attached to the sample holder.

FTIR patterns were obtained from a Frontier FT‐IR Spectrometer (PerkinElmer).

Water contact angle measurements were performed by pipetting 5 µL of Milli‐Q water onto the CNC films and photographing the samples after 10 s. The photographs were then analyzed using Drop Analysis plugin (under LB‐ADSA method) of ImageJ (https://bigwww.epfl.ch/demo/dropanalysis/) to measure the water contact angles.

TGA was performed on a TG 209 F1 Libra thermogravimetric analyzer (NETZSCH), with a sensitivity of 0.1 µg. All tests were conducted under nitrogen with a flow rate of 50 mL min^−1^ and programmed for data collection between 30 and 800 °C, with a heating rate of 10 °C min^−1^.

PXRD patterns were collected using an Empyrean expert multipurpose X‐ray diffractometer (Malvern Panalytical) with a Cu LFF HR sealed tube X‐ray source and a 1D PIXcel3D detector, operating at 45 kV and 40 mA in Bragg–Brentano configuration.

^13^C CP‐MAS solid‐state NMR measurements were performed on a Varian Unity INOVA 400 spectrometer with a Varian/Chemagnetics 3‐channel 4 mm T‐3 MAS probe. Samples were packed in a 4 mm zirconia rotor that was sealed with a Teflon cap and then spun at 5 kHz at room temperature. Spectra were collected with rf field strengths of 83 kHz for CP, a 1 ms contact time, 60 kHz continuous ^1^H decoupling, and a recycle delay between 3 to 4 s. Each spectrum was the result of 20000 scans. The reported ^13^C chemical shifts were externally referenced to glycine (carbonyl group set at 176.03 ppm).

## Conflict of Interest

The authors declare no conflict of interest.

## Author Contributions

Z.L. conceived the project, performed the synthesis of the materials and their characterizations, wrote the manuscript and supporting information. P.W. helped with materials fabrication and characterization, and manuscript preparation. Y.Z. and C.A.M. performed all solid‐state NMR experiments. M.J.M. did the supervision, manuscript revision, and funding acquisition.

## Supporting information



Supporting Information

## Data Availability

The data that support the findings of this study are available from the corresponding author upon reasonable request.
