# Supplementary material for: Durable Hydrophobic Iridescent Films with Tunable Colors from Self‐Assembled Cellulose Nanocrystals
Source: Small. 2024 Dec 24;21(6):2409701. doi: 10.1002/smll.202409701 (PMC11817899; doi:10.1002/smll.202409701)
Supplement: Supplementary file 1 — Supporting Information [file SMLL-21-2409701-s001.pdf]

NANO · MICRO
small

Supporting Information

for *Small*, DOI 10.1002/smll.202409701

Durable Hydrophobic Iridescent Films with Tunable Colors from Self-Assembled Cellulose Nanocrystals

*Zongzhe Li, Phoebe Wang, Yinghao Zhang, Carl A. Michal and Mark J. MacLachlan**

Supporting Information

Durable Hydrophobic Iridescent Films with Tunable Colors from Self-Assembled Cellulose Nanocrystals

*Zongzhe Li, Phoebe Wang, Yinghao Zhang, Carl A. Michal and Mark J. MacLachlan**

S1. Supporting Methods

S1.1. Calculation of Saturated Solvent Contents (Q_s)

In a typical solvent absorption - desorption tests, a piece of freshly made CNC-SDFR-C_9 film was transferred into a pre-weighed 20 mL glass scintillation vial, and weighed together with the vial to get its own weight (m_0). The solvent absorption was performed by soaking the film in the desired solvent for at least 2 h to guarantee full saturation. The solvent was then removed using syringe and needle, and the swollen film was weighed together with the vial to measure the weight of absorbed solvent (m_s). The solvent desorption was performed by air drying the material in the fume hood overnight. Finally, the recovered weight was also calculated based on the weight of the recovered film together with the vial. Then the saturated solvent content (Q_s) can be calculated based on the following Equation (1):

$$Q_s = \frac{m_s}{m_0} \quad (1)$$

S1.2. Calculation of Degree of Substitution (DS)

In order to calculate the degree of substitution (DS), the percentage of functionalization (PF) based on carbon weight was first calculated using the following Equation (2):

$$PF = \frac{C_C - C_0}{C_T - C_C} \quad (2)$$

where C_0 is the carbon content of CNC-SDR, C_C is the carbon content of the tested functionalized film, and C_T is the theoretical carbon content of the alkyl chain (including the carbonyl group) of the corresponding acid anhydride being used for the functionalization.

And then, the DS can be calculated from PF using the following Equation (3):

$$DS = \frac{PF*6}{N_C} \quad (3)$$

where N_C is representing the number of carbons of the alkyl chain (including the carbonyl group), and 6 is corresponding to the number of carbons in the repeat unit of cellulose.

S2. Supporting Figures and Tables

S2.1. Chiral Nematic Glucose-CNC (g-CNC) Films

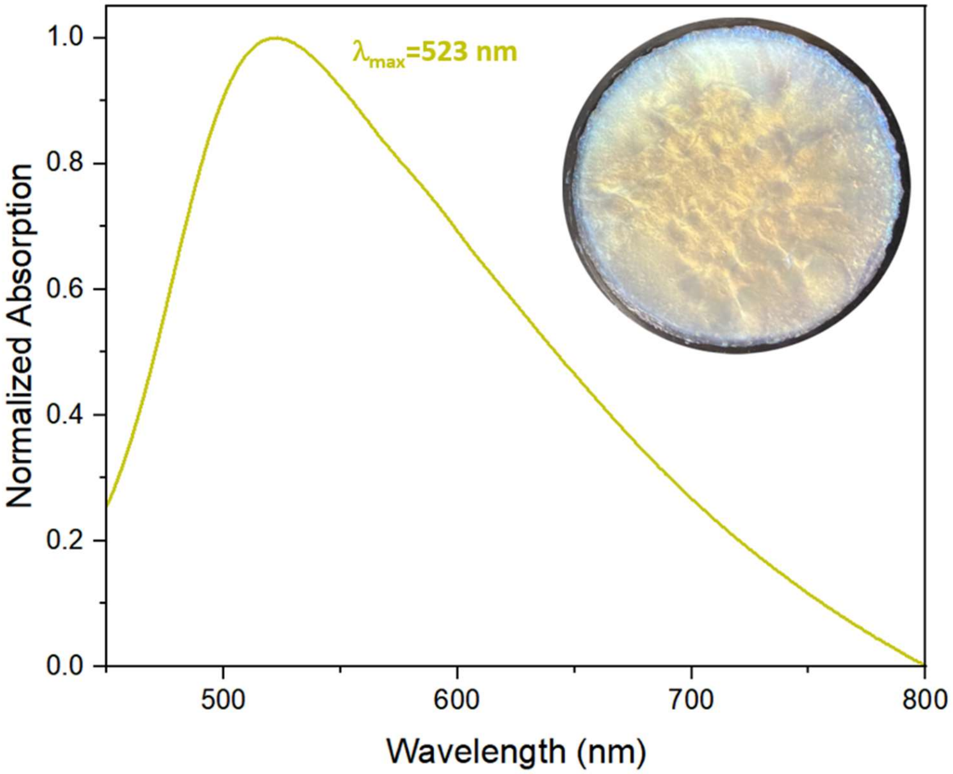

Figure S1. UV-vis spectrum of the g-CNC film. Inset: physical appearance of the g-CNC film (diameter: 48 mm). The inset photograph was taken under ambient light without polarizers while the sample was resting on a black cloth.

S2.2. Immersion of the g-CNC Films

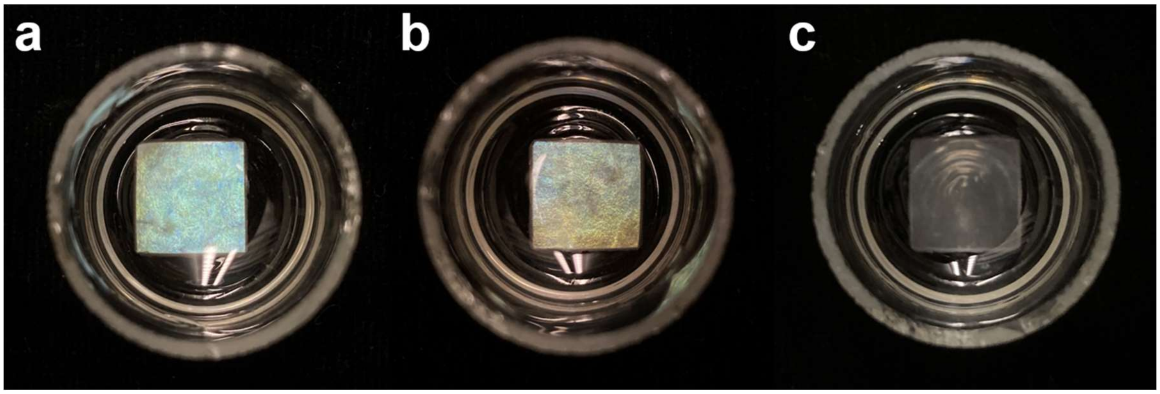

Figure S2. Images of g-CNC film soaking in DMSO after (a) 0 min, (b) 5 min, and (c) 40 min. (film size: 1 × 1 cm). The photographs were taken under ambient light without polarizers.

As shown in Figure S2, the g-CNC film presents a gradually red-shifting color as it is soaked in DMSO, reaching a colorless swollen state after 40 min. This color change is due to the infiltration of DMSO into the chiral nematic CNC matrix (containing glucose), which increases the helical pitch.

S2.3. Elemental Analysis of CNC Films

Table S1. Content of Carbon and Hydrogen, and degree of substitution (DS) of CNC films.

Sample	Content of C [%]	Content of H [%]	DS
g-CNC	41.19	6.26	--
CNC-SDR	42.71	6.35	--
CNC-SDFR-CMe_3	45.32	6.43	0.12
CNC-SDFR-C_3	46.87	6.48	0.30
CNC-SDFR-C_6	49.60	7.02	0.24
CNC-SDFR-C_9	51.08	7.21	0.19

As glucose has the same chemical composition as cellulose, the loss of glucose should not lead to any changes in the carbon and hydrogen content. However, the CNC-SDR showed a slightly higher content of carbon and hydrogen compared to g-CNC. Although counterintuitive at first glance, this can be rationalized by the hydrothermal desulfation of the CNCs, during which the loss of sulfate half-ester groups leads to a lower content of oxygen and sulfur, subsequently resulting in slightly higher contents of carbon and hydrogen in CNC-SDR. After functionalization, the grafting of carbon chains leads to a higher content of carbon and hydrogen compared to CNC-SDR. In addition, the content of carbon and hydrogen increases along with the increase in the length of grafted carbon chains, which is attributed to their larger proportion of carbon and hydrogen atoms. It's noteworthy that CNC-SDFR-CMe_3 has one more carbon and two more hydrogen atoms in its carbon chain compared to CNC-SDFR-C_3, but still shows a slightly lower content of carbon and hydrogen. This may be due to its greater steric hindrance, which leads to a lower grafting extent on the surface of CNCs.

S2.4. FTIR Analysis of CNC-SDFR-C_9

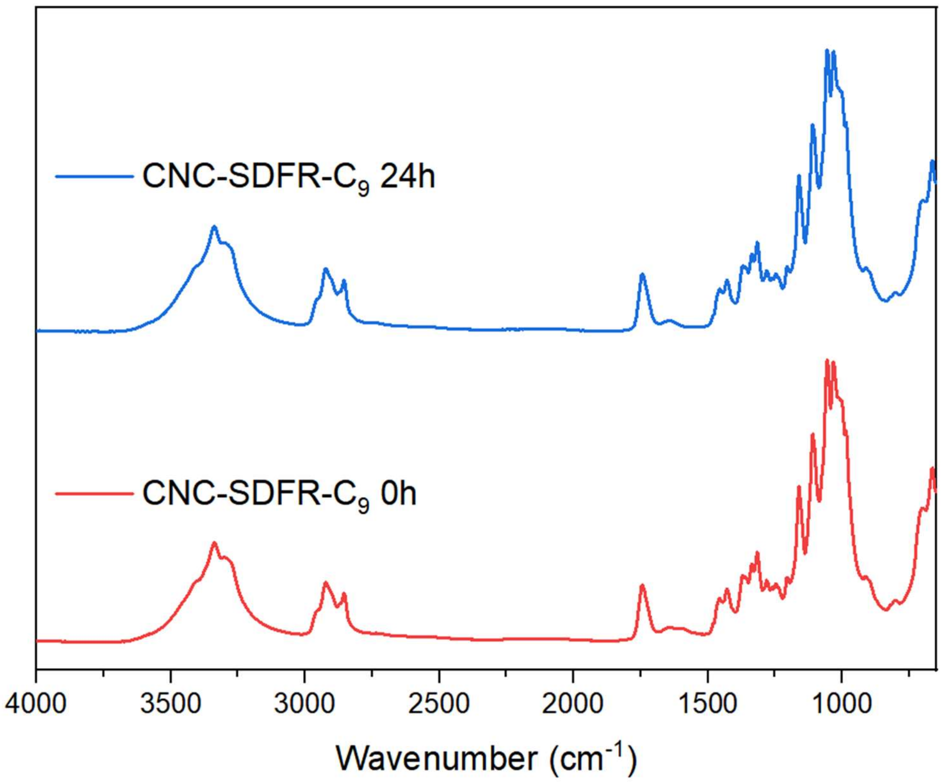

Figure S3. FTIR spectra of CNC-SDFR-C_9 before (0 h) and after soaking in water for 24 h.

S2.5. Solvent Absorption – Desorption Cycling Tests

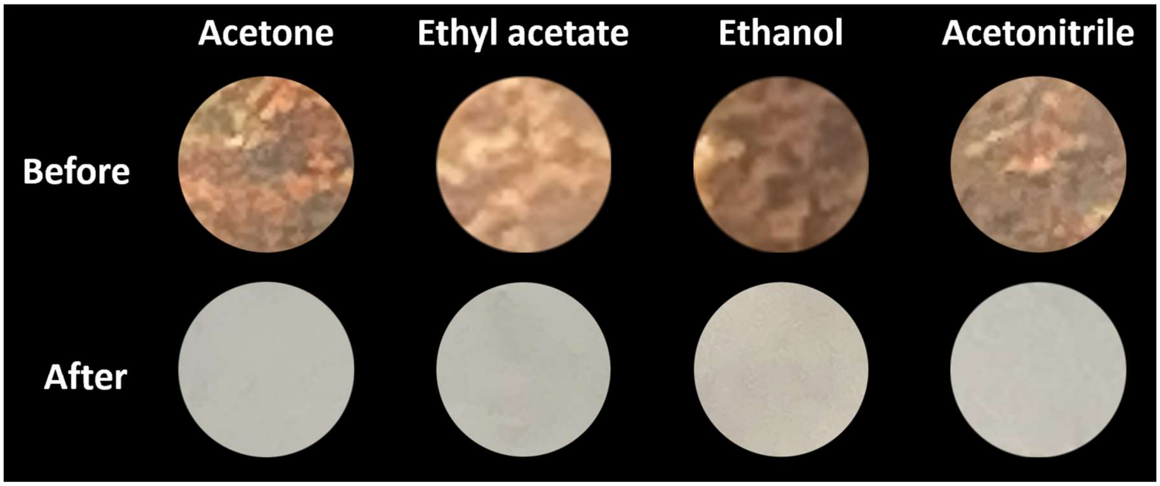

Figure S4. Photographs of CNC-SDFR-C_9 before and after saturation with various solvents. The absorption of solvents increases the helical pitch of the chiral nematic structure, thus red-shifting the original red color to the infrared region, resulting in colorless materials. The samples shown here are 10 mm in diameter. Photographs were obtained under ambient light without polarizers, cropped into a circular shape and superposed on a black background. The samples in the top row were photographed on a black cloth while those in the bottom row were photographed on white paper.
